# Synaptic augmentation in a cortical circuit model reproduces serial dependence in visual working memory

**DOI:** 10.1371/journal.pone.0188927

**Published:** 2017-12-15

**Authors:** Daniel P. Bliss, Mark D’Esposito

**Affiliations:** 1 Helen Wills Neuroscience Institute, University of California, Berkeley, CA, United States of America; 2 Department of Psychology, University of California, Berkeley, CA, United States of America; University of California San Diego, UNITED STATES

## Abstract

Recent work has established that visual working memory is subject to serial dependence: current information in memory blends with that from the recent past as a function of their similarity. This tuned temporal smoothing likely promotes the stability of memory in the face of noise and occlusion. Serial dependence accumulates over several seconds in memory and deteriorates with increased separation between trials. While this phenomenon has been extensively characterized in behavior, its neural mechanism is unknown. In the present study, we investigate the circuit-level origins of serial dependence in a biophysical model of cortex. We explore two distinct kinds of mechanisms: stable persistent activity during the memory delay period and dynamic “activity-silent” synaptic plasticity. We find that networks endowed with both strong reverberation to support persistent activity and dynamic synapses can closely reproduce behavioral serial dependence. Specifically, elevated activity drives synaptic augmentation, which biases activity on the subsequent trial, giving rise to a spatiotemporally tuned shift in the population response. Our hybrid neural model is a theoretical advance beyond abstract mathematical characterizations, offers testable hypotheses for physiological research, and demonstrates the power of biological insights to provide a quantitative explanation of human behavior.

## Introduction

Standard paradigms for measuring the contents of visual working memory have revealed that human observers tend to merge features of stimuli from previous trials into their representation of the current one, leading to a systematic bias in behavioral reports [1–15]. This smoothing of representations—termed “serial dependence”—is spatiotemporally tuned [1–7, 12, 14–16] and sensitive to the featural similarity between items [1–3, 7–10, 12, 14, 15, 17, 18]. While it has been proposed that serial dependence directly alters stimulus perception [1, 8]—and precedes the onset of memory or decision making [1, 8]—more recent studies have demonstrated that the trial-history bias is absent at the time of perception [9, 15] and evolves slowly during the subsequent delay period of a working memory task [9, 15, 17, 18], reaching an asymptote around six seconds after the most recent item has been encoded into memory and removed from view [15]. This suggests that serial dependence may be more associated with working memory maintenance than with perceptual encoding. The working memory system tends to preferentially maintain the objects in visual scenes toward which observers direct their attention [19]. In natural environments, the focus of attention tends to track the same object for several seconds at a time [20]. This implies that the contents of visual working memory do not shift radically from moment to moment. Hence, a temporal smoothing operation like serial dependence would seem to be a useful mechanism for maintaining the stability of the current representation [9, 15, 21].

The evidence that serial dependence in behavior is more strongly associated with the contents of working memory than immediate perception provides a starting point for the investigation of its neural mechanisms. To date, theories of working memory representation in cortex have not sufficiently accounted for serial dependence. Furthermore, the neural code that supports working memory in general (serial dependence aside) has been the subject of considerable debate in the past few years [22–27]. A classical view [24, 25, 28], inspired by pioneering work in the Goldman-Rakic Lab [29], is that persistent activity in cortical neurons, tuned to stimulus features, sustains representations across memory delay periods between perception and action. Reverberatory mechanisms have been posited to support persistent activity [30–32]. Recently, alternative theoretical considerations [33], as well as occasional failures to identify persistent activity in physiological experiments [34–37], have called this account into question. A newer “activity-silent” [27] model proposes that a brief burst of neuronal firing may be sufficient to drive synaptic changes in cortical networks that can then maintain the information in memory in the absence of continued elevated firing rates [22, 23, 26, 33]. This theory relies on the finding that the capacity for short-term synaptic plasticity—in particular, synaptic augmentation—is enriched in prefrontal cortical areas associated with working memory [38, 39].

Preliminary attempts to demonstrate how existing (or revised) neural models of working memory would give rise to serial dependence have focused on an activity code, rather than changes in synaptic weights [17]. It has since been argued that accounting for dynamic synapses might reconcile discrepant trial-history effects in neural recordings and behavior [18], but the biophysical details of how this would work have not been modeled. Perceptual studies have shown how short-term plasticity could explain a shift in responses from one trial to the next [40, 41], but these studies have focused not on the blending that occurs during serial dependence, but on the opposite, repulsive shift associated with sensory adaptation. Adaptation and serial dependence manifest as mirror-opposite biases in behavior, but, unlike serial dependence, adaptation has been found to be independent of attention, which suggests it may be a phenomenon of passive perception rather than working memory [1, 9]. Plasticity occurs at many time scales in the brain, from synaptic facilitation, which returns to baseline within hundreds of milliseconds, to homeostatic scaling that can last for hours or days [42]. The time scale of the rise and fall of serial dependence in behavior (∼10 s [15]) is similar to that of synaptic augmentation—the form of plasticity observed to be especially enriched in prefrontal neural circuits important for working memory [38, 39]. Synaptic augmentation is an increase in synaptic vesicle release that occurs when calcium accumulates in presynaptic terminals [43] in direct proportion to the presynaptic firing rate [38].

In the present study, we investigate whether biophysically-detailed models of working memory storage can quantitatively account for the spatiotemporal tuning of serial dependence in memory-guided behavior. The modeling framework we use for this analysis is the “bump attractor” framework for the cortical representation of continuous visual features in working memory [32, 44]. Bump attractor dynamics provide a close match not only to human behavioral performance [45, 46], but also to the physiology and anatomy of prefrontal cortex [32, 47, 48]. For this reason, we considered this modeling platform a suitable testing ground for competing theories of the biophysical basis of serial dependence. In the bump attractor model, it is proposed that active neural firing sustains memories. Individual excitatory neurons in the model network are tuned to task-relevant stimulus features, and stimulus-driven activity persists in the network over the working memory delay period as a result of synaptic reverberation that depends on NMDA receptors [30]. The stability of the network’s response also depends on broad lateral inhibition from local interneurons and a balance of excitatory and inhibitory signaling. Numerous empirical studies spanning several decades support the importance of these prefrontal network features for working memory function [29, 31, 47–49].

First, we demonstrate that the classical bump attractor model is insufficient to account for the temporal dynamics of serial dependence. This is true even when the model is adjusted to allow the population response for each trial to persist into the intertrial interval (ITI) and disrupt encoding of the subsequent stimulus—an “active firing” mechanism for the trial-to-trial interference that serial dependence represents. In contrast, endowing synapses in the bump attractor network with the capacity for realistic synaptic augmentation [38, 39, 50] causes a gradual potentiation of serial dependence over the memory delay period that asymptotes within 10 s, consistent with human psychophysics [15]. Furthermore, between trials, in the absence of new visual input, serial dependence in the simulated circuit decays within 10 s, again matching human behavior [15]. Importantly, our integrated model with short-term plasticity represents a hybrid of principles from traditional and “activity-silent” theories of working memory, demonstrating with computational precision how these competing accounts can co-exist in the same circuit and make dissociable contributions to memory-guided behavior.

## Results

### Model 1: Original bump attractor model (with fixed synapses)

**Fig 1:**
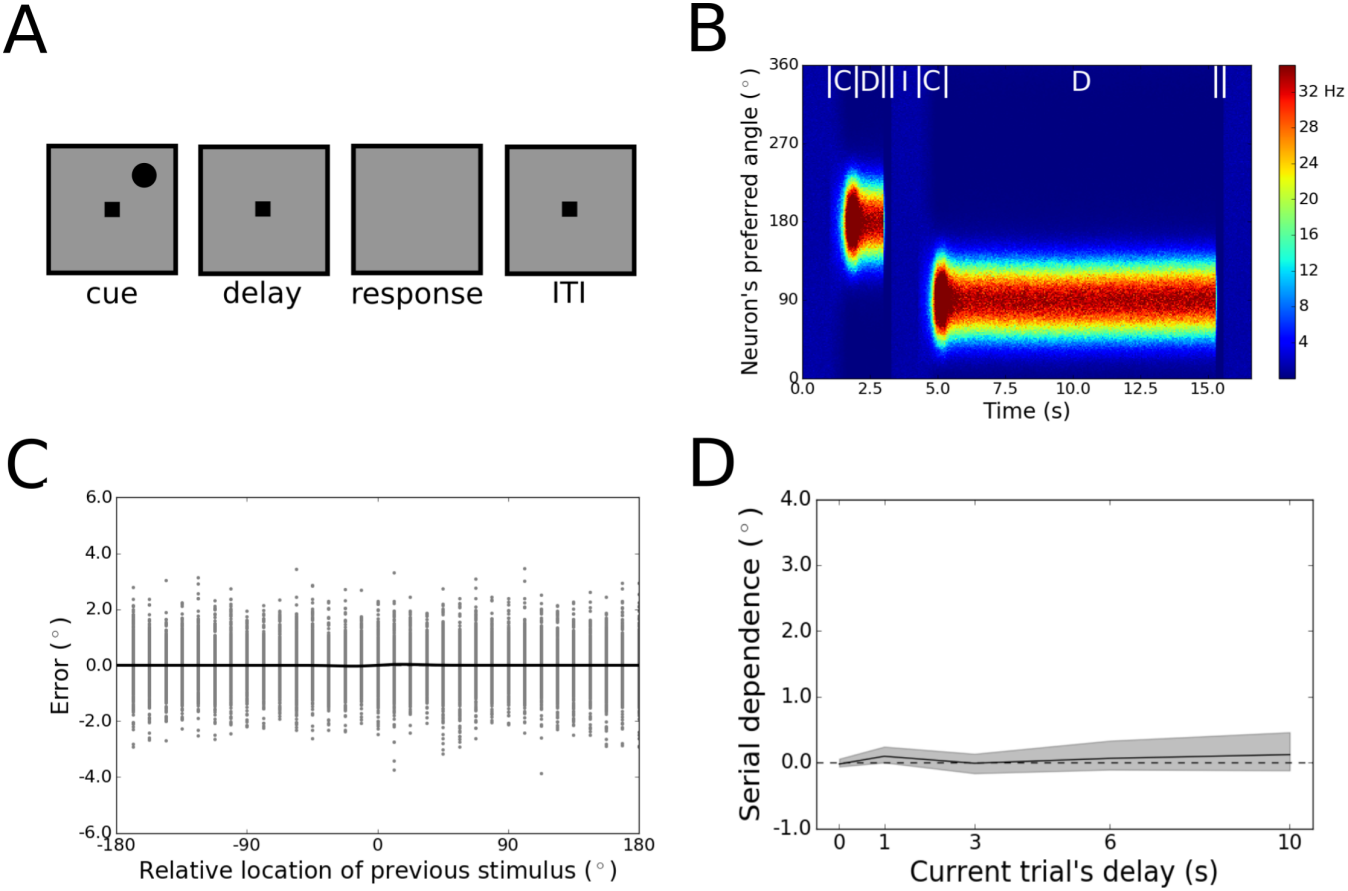
Results for Model 1. (A) The events in each trial of the working memory task used to test Models 1-3. Each trial started with the presentation of a cue whose angle from fixation was varied. Cue presentation was instantiated as an injection of current into the neurons, with the magnitude of the current set as a Gaussian function of the difference between the stimulus feature value and each neuron’s preferred location. The first cue in each pair of trials was presented at 180°. One of 32 angles evenly spaced around 360° was chosen for the second stimulus. The cue was maintained in the network across a delay period (without continued stimulus-driven current injection). Finally, the removal of the fixation square from the screen signaled the start of the response period, when the network’s response was decoded and activity was reset via the passage of unstructured inhibitory current into the network. An ITI separated each response period from the subsequent cue period. (B) Activity in the network, with neurons lined up along the y-axis according to the stimulus angle to which they respond most strongly. Firing rates are color-coded. Two trials are shown with different delay lengths, the first with the stimulus at 180° and the second with the stimulus at 90°. At the start of each trial, during the cue period (labeled C in the figure), a bump of activity forms in the network, centered at the stimulus location. This bump weakens slightly during the transition to the memory delay period (labeled D), but is maintained throughout the delay. The bump is reset at the end of each delay, when the response is made. After the response, the ITI (labeled I) begins. (C) Tuning of serial dependence across all angular differences between the current and previous stimulus. Raw errors for every simulation are in gray, and the best fit of the derivative of Gaussian (DoG) function used to model serial dependence is in black. No serial dependence is apparent in the responses—error is distributed evenly around a horizontal line at 0°. (D) Peak-to-peak of the DoG function across the working memory delay. Shading depicts bootstrapped 95% confidence intervals. There is no evidence of serial dependence regardless of the duration for which the network maintains the current trial’s stimulus feature.

First, we confirmed that the traditional bump attractor model [32, 44] does not produce a signal that corresponds to serial dependence in human memory-guided behavior. In order to do this, we ran simulations of a standard working memory delayed response task through the network (Fig 1A). The generic task structure we used is common in studies of serial dependence [1, 2, 8, 9, 15, 17, 18]. On each trial, a stimulus is presented briefly and then removed. A feature of this stimulus must be remembered over a blank delay period (usually a few seconds in length). Finally, the end of the delay is signaled, and a response is made to indicate the feature value in memory. In Fig 1A, the task-relevant visual feature depicted is the stimulus’ spatial location. However, the model does not depend on this interpretation. Serial dependence in human behavior has been observed to occur for a wide variety of feature types [21], and the bump attractor model can accommodate all continuous feature spaces equally well.

A simulated behavioral response is read out of the network’s activity at the end of the delay period of each trial using population vector decoding [51]. The mechanism of mnemonic maintenance in the model is sustained, elevated firing among excitatory neurons tuned to the task-relevant visual feature. Decoding is possible because, across the network as a whole, the preferred locations of the individual neurons evenly tile the feature space. The population response of the network can be visualized as a self-sustaining bump of activity, centered at the visual feature value encoded at the start of the trial (Fig 1B)—the center of this bump is what is decoded as the network’s behavioral output.

We implemented the reduced firing-rate version of the model [44], given that it recapitulates the behavior of the full spiking model [32] while consuming far fewer computer processing resources [52]. In this reduced version, the equations of the model are used to compute an instantaneous firing rate at each time point for each neuron in the network, whereas in the full version of the model, the occurrence of every individual action potential is computed. Both versions include noisy input to the network from other (unmodeled) cortical populations whose neurons are not tuned to the task-relevant stimulus feature. The recurrent connectivity profile within the network is such that neurons are most strongly connected to those of their neighbors that are tuned to similar locations in feature space, with the magnitude of the coupling strength between any pair of neurons set as a Gaussian function of the difference between their preferred locations. The simulations we ran each comprised a single pair of trials (Fig 1B), with different combinations of stimulus values used for different trial pairs. Thirty-two combinations of stimuli were used in total, with the angular difference between consecutive stimuli varied uniformly around the circular feature space. For each combination of trials, 100 simulations with different random seeds were performed.

As expected, despite realistic levels of noisy firing in the network, the behavioral performance of the model was on average veridical, with no systematic bias in responses relative to the stimulus from the previous trial (Fig 1C). This result is not consistent with human [1, 2, 9, 15] and non-human [17, 18] primate performance on this task. The absence of serial dependence in the bump attractor model is not specific to any particular moment in the delay period. When the network was probed to make a response at variable time points after stimulus offset, there was no detectable increase or decrease in the trial-history bias—serial dependence was near zero throughout (Fig 1D).

### Model 2: Activity leak model (with fixed synapses)

The null results presented above provided an impetus for making adjustments to the bump attractor model to allow serial dependence to be observed. It has been proposed—within the traditional rate code model of working memory—that persistent activity may extend beyond the end of a given trial and leak into the next one, causing a disruption of the encoding of the subsequent stimulus that depends on the distance between the successive activity bumps [17, 18, 21]. This leak of activity that encodes one stimulus value and disrupts the encoding of the next stimulus value would be a potential mechanism for the serial dependence observed behaviorally. There is evidence from data collected from the frontal eye fields of non-human primates that elevated neural firing does persist from one trial of a delayed-response task into the next [18]. However, it is unknown whether this activity leak biases neural responses in the way that can explain the pattern of serial dependence in behavior [18].

**Fig 2:**
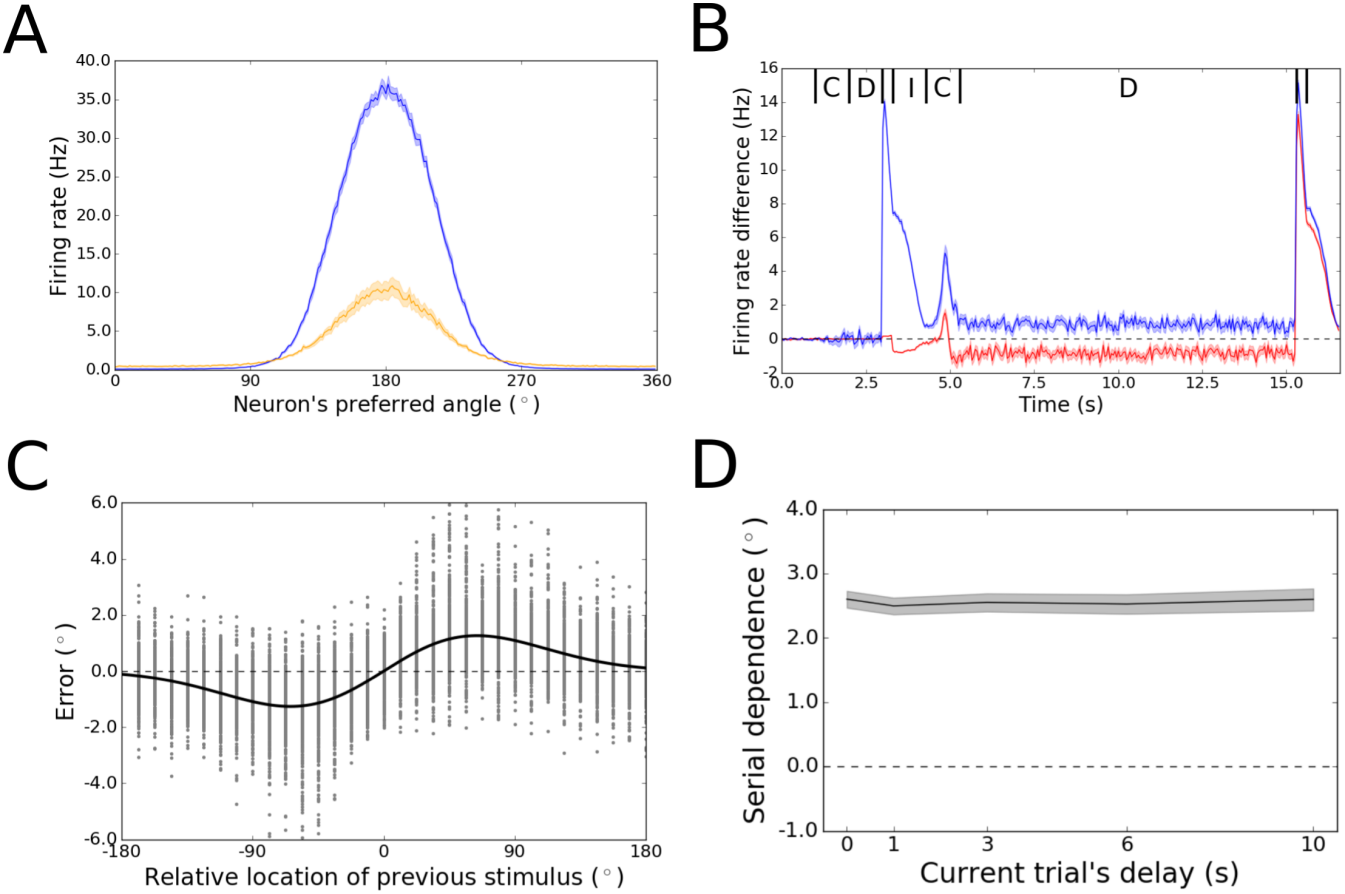
Results for Model 2. (A) Consistent with physiological data from monkeys [18], the amplitude of the activity bump during the ITI (yellow) is approximately one-quarter the size of the bump in the middle of the working memory delay (blue) in the activity leak model. Shading indicates the standard error of the mean for a single representative trial. (B) Difference between the activity patterns in the activity leak model and the original bump attractor model for two example neurons (blue and red). Shown are the firing rates for these two neurons in the activity leak model subtracted by their firing rates in the original model. A single pair of trials is displayed, just like in Fig 1B. In this case, the first stimulus was presented at 180° and the second at 112.5°. The two neurons shown have preferred angles of 146.25° (blue) and 78.75° (red), which places them on opposite flanks of the bump formed for the second stimulus in this pair. The two models perform equivalently for the cue and delay periods (labeled C and D) of the first trial. However, activity during the first response and subsequent ITI (labeled I) for the neuron in blue is much higher in Model 2 than in Model 1, because the bump from the first trial persists during these periods only in Model 2. (In addition to being on a flank of the bump for the second stimulus in this pair, the neuron in blue is also on a flank of the bump for the first stimulus at 180°—see Fig 1B.) The residual activity dissipates noticeably during the ITI, but is sufficient to bias activity during the cue period of the second trial, such that the firing rates for the two neurons are shifted away from their rates in Model 1 by a few Hz in either direction. The directionality of this shift in firing rates indicates that the bump on the second trial has shifted toward the previous stimulus. (C) Tuning of serial dependence across angular differences between consecutive stimuli. Raw errors are in gray, and the best fit of the DoG function is in black. Serial dependence with an amplitude consistent with what is observed in humans is apparent. (D) Peak-to-peak of the DoG model across the working memory delay period. Shading represents bootstrapped 95% confidence intervals. Serial dependence is non-zero at all delays. The amplitude of serial dependence is large at the moment of stimulus offset and does not change over time.

We implemented activity leak in the bump attractor model by weakening the mechanism that resets the network during the response period of each trial. Specifically, in the default version of the model, the start of the response period causes widespread inhibition in the network that shuts off the tuned bump of activity and returns the neurons to unstructured, baseline levels of firing before the start of the next trial. We reduced the inhibitory current passed into the network at the response period by 88.4%. This allowed the network to obtain levels of leak firing into the inter-trial interval (ITI) that are commensurate with observations in monkey cortex [18] (Fig 2A).

Again, 100 simulations of the task with different random initial conditions were executed for each of 32 combinations of trial pairs, with the differences between successive stimuli uniformly covering the range of possible differences. The leak of elevated firing into the ITI in this version of the model is evident in Fig 2B, which shows the difference between Model 1 and Model 2 in the firing rates of two example neurons during one pair of trials. The blue trace in this figure corresponds to a neuron with preferred angle 146.25°, which is on the flank of the bump of activity for the first stimulus presented, at 180°. During the ITI after this first trial, this neuron’s activity remains elevated relative to its activity in Model 1, and encoding of the next trial’s stimulus is shifted as a function of this residual firing. The red trace corresponds to a neuron whose preferred angle is far from the first stimulus, at 78.75°, and whose activity does not change much during the ITI. The shift in the encoding of the second stimulus (at 112.5°), however, is reflected in the firing rates of both depicted neurons, as they fall on opposite flanks of the activity bump that forms on the second trial. The decreased firing rate for the neuron at 78.75° (red) and increased firing rate for the neuron at 146.25° (blue) during the second trial’s delay period indicate that the bump has shifted in the direction of the preceding trial’s stimulus (180°).

Across all trial pairs, the activity leak model, unlike the original bump attractor model, gave rise to a pattern of serial dependence that depended on the angular difference between the consecutive stimuli. When consecutive bumps were far apart or at identical locations, there was negligible disruption of encoding. However, in an intermediate range of stimulus differences, the merging of the residual bump from the previous trial with the newly forming bump of the current trial shifted the new bump from the correct feature value by a few degrees. The distance dependence of this merging effect caused Model 2 to produce a pattern of decoded behavioral responses that bear a striking resemblance to what has been observed in the behavior of humans [1, 2, 9, 15] and monkeys [17, 18] (Fig 2C). The same function that has been used to fit the tuning of serial dependence in human behavior [15] – the derivative of Gaussian (DoG)—provides an excellent fit to the tuning over stimulus differences from the activity leak model. Furthermore, the amplitude of the fit (2.53° peak-to-peak) is on the order of that for the human data (3.37° peak-to-peak for the same timing of task events [15]). However, over the full range of working memory delay periods tested—from 0 s (extraction of the behavioral response immediately after perceptual encoding) to 10 s—the amplitude of serial dependence in the activity leak model remained constant (Fig 2D). This is in stark contrast with human performance, which shows a gradual rise in the magnitude of serial dependence as the memory delay advances – for up to 6 s [15].

The failure of Model 2 to reproduce the temporal dynamics of serial dependence (despite reproducing its tuning over angular differences between consecutive stimuli) can be explained upon consideration of the activity leak mechanism. Residual firing from the previous trial interacts with the newly forming bump at just one moment in time – the start of the second trial in each trial pair. After that, the center of the new bump is established (shifted slightly from the stimulus’ true feature value), and the residual activity is quashed (as in primate cortex [18]). In the model, this erasure of the residual activity upon the commencement of the new trial’s cue period is a consequence of two factors: spontaneous dissipation of the residual activity during the ITI (visible in the blue trace of Fig 2B) and lateral inhibition driven by the new stimulus once the second cue period begins. Once the residual activity has either returned to baseline or been incorporated into the second trial’s bump of activity during the cue period, no mechanism remains to systematically alter the new bump’s drift as the delay proceeds—hence, the level of serial dependence remains constant.

Our results for Model 2 establish that leak activity at levels observed in primate cortex [18] cannot explain the scaling with working memory delay of serial dependence in human behavior. Still, our results do not rule out that stronger levels of leak activity—sufficient to overcome lateral inhibition during the subsequent cue period—might provide a good match to the human data. To resolve this possibility, we ran additional simulations with the model parameters set such that the bump of activity from the first trial in each trial pair did not diminish at all during the response period of that trial and persisted at full strength into the subsequent ITI and cue period. That is, instead of reducing the response-related inhibitory current passed into the network by 88.4%, we now reduced it to zero. Even with this much stronger influence of activity from the previous trial on the formation of the bump in the new trial, the model failed to recapitulate the temporal evolution of the behavioral effect observed empirically—once again, the interaction between the successive activity bumps was limited to the cue period of the second trial in each trial pair, and after that the magnitude of the shift in the new bump location remained fixed, on average (S1 Fig). Furthermore, the tuning of the serial dependence that occurred in the network no longer followed the DoG shape typical of human behavior. Instead, the effect was nearly linear over angular differences between consecutive stimuli – such that the network’s responses were centered around the previous trial’s feature value and information about the current trial was lost completely (S1 Fig).

### Model 3: Bump attractor model with plastic synapses

Neither the original bump attractor model nor the activity leak version could reproduce all of the temporal dynamics of serial dependence in human memory-guided behavior. Hence, we turned to a property of neural circuits that has been assigned a great deal of importance in newer theories of working memory storage: short-term synaptic plasticity [22, 23, 26, 27, 33]. In particular, we focused on synaptic augmentation [43], both because its rate of accumulation and decay [38, 39, 43] matches the time scale of serial dependence [15], and because it is a prominent synaptic dynamic in prefrontal cortical circuits that are associated with working memory [38, 39]. The magnitude of augmentation is a function of calcium dynamics in presynaptic terminals [43]. Each action potential passing into the terminal increases the likelihood that calcium channels are open. In the absence of spiking, intracellular calcium levels decay to baseline exponentially [43]. The dynamics of synaptic weight updates due to these processes has been implemented successfully in phenomenological neural-network models that do not include explicit variables for voltage-gated calcium channels or individual action potentials [50, 53]. Consistent with these theoretical models, as well as empirical work [38, 39, 43], we modeled the rise of synaptic augmentation for a given synapse as being proportional to the presynaptic firing rate (up to a saturation point) and assumed exponential decay. The capacity for augmentation was instantiated for every synapse in the original bump attractor network. (Because synaptic depression often co-occurs with augmentation [38, 39]—but with a shorter time constant—we implemented depression alongside augmentation, as has been done previously [50, 53]. In our model, augmentation is the dominant dynamic.)

**Fig 3:**
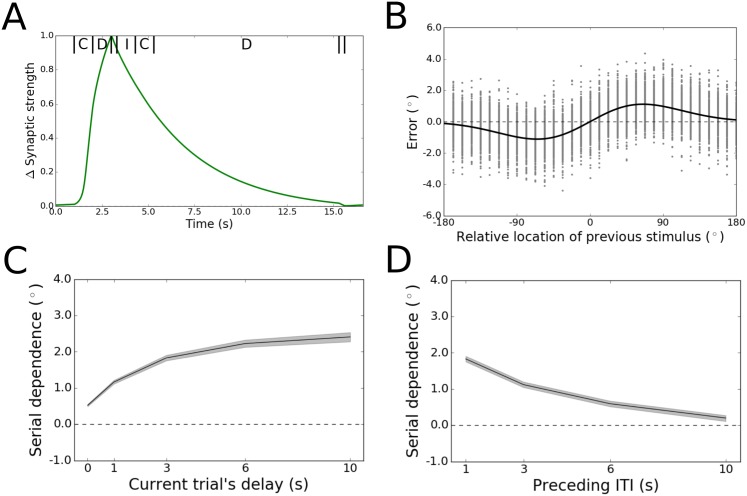
Initial results for Model 3. (A) Normalized mean change in synaptic strength (relative to the pre-stimulus baseline) for efferent connections from a sample neuron with preferred angle 213.75°. Average results for a single pair of trials are displayed. For this trial pair, the first stimulus was presented at 180° and the second at 112.5°. Synaptic augmentation causes the connection strengths to increase during the first trial’s cue (labeled C) and delay (labeled D) periods. During the first reponse period, the highlighted neuron’s firing rate returns to baseline levels (Fig 1B) and its efferent connection strengths slowly return to their original values over the next several seconds, according to the time constant of augmentation (4.2 s). The synaptic strength shown is the *F* parameter of the model, normalized to span the range (0, 1). (B) Tuning of serial dependence across angular differences between consecutive stimuli. Raw errors are in gray, and the best fit of the DoG function is in black. Serial dependence with an amplitude consistent with that observed in humans is apparent. (C) Peak-to-peak of the DoG fit across the working memory delay. Shading represents bootstrapped 95% confidence intervals. Serial dependence increases gradually over time before reaching an asymptote within 10 s. (D) Peak-to-peak of the DoG fit across different ITIs. When the ITI is increased from 1 to 10 s, serial dependence becomes monotonically weaker.

We again ran 100 simulations of the task for each of 32 trial pairs, uniformly sampling the range of possible trial-to-trial stimulus differences. Fig 3A shows how synaptic strengths change across one of these trial pairs for a sample neuron. Illustrated is the average change in connection strengths from the neuron whose preferred angle is 213.75° to all other neurons in the network. When the first stimulus is presented at 180°, synaptic augmentation causes the connection strengths to increase. They reach their peak at the end of the trial, and then gradually return to baseline over the course of the subsequent trial (whose stimulus is presented at 112.5°). Like the activity leak model (Model 2), the augmentation model produced behavioral responses that tracked the pattern of serial dependence over stimulus differences seen in humans and monkeys (Fig 3B, 2.23° peak-to-peak). We observed that the width of the tuning of the trial-history effect was sensitive to the width of the tuning of individual neurons in the network (see Materials and methods). Wider neuronal tuning curves widened the tuning of serial dependence—as this caused augmented synapses from the previous trial and the bump of activity on the new trial to interact over greater distances in the network—whereas narrower tuning of individual neurons narrowed the spread of serial dependence (S2 Fig).

Most important with regard to the key shortcoming of the activity leak model, the augmentation model gives rise to a slowly evolving time course of serial dependence over the working memory delay that quantitatively matches psychophysics data (Fig 3C). For each of the tested delay lengths between 1 and 6 s after stimulus offset, the amplitude of the history effect exceeded the confidence bounds of the next-shortest delay. Also consistent with the human data [15], serial dependence reached an asymptote in the network after 3 s and before 10 s. Furthermore, its final amplitude at 10 s falls within the confidence bounds of the human data for this delay length [15]—making the model a tight fit to human behavior across the full range of working memory delay periods that has been tested.

We confirmed that this time course in the model is specific to the memory period, and not an artifact of the mere passage of time independent of maintenance demands. To do this, we extended the ITI between the trials within each pair, keeping the delay length constant. We ran 100 simulations with different random seeds for each of 32 stimulus differences and four ITIs. Serial dependence was inversely proportional to ITI length (Fig 3D). Again, this result precisely matches human behavior [15].

**Fig 4:**
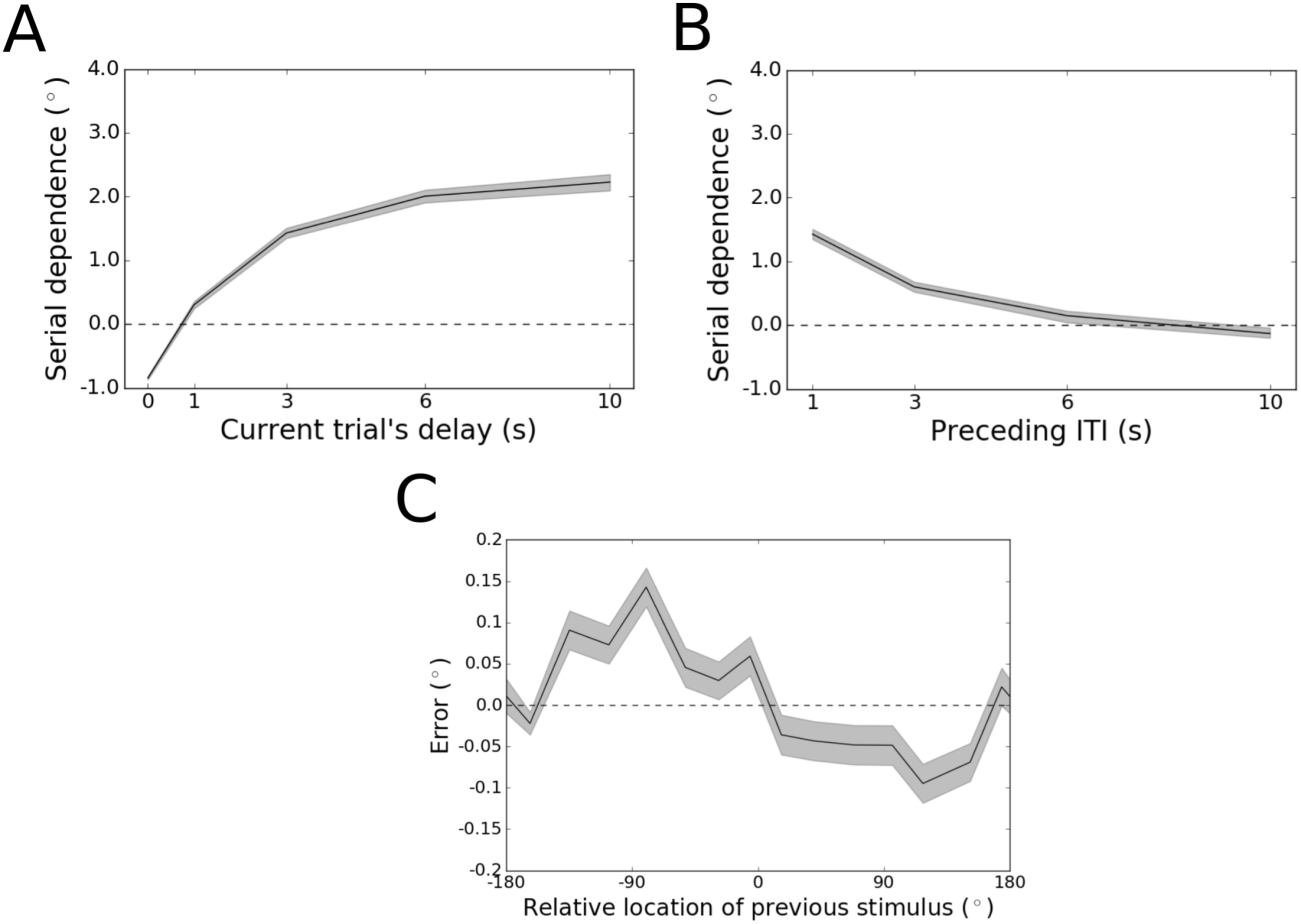
Results for Model 3 after accounting for adaptation. (A) Peak-to-peak of the DoG fit across the working memory delay. Shading represents bootstrapped 95% confidence intervals. Immediately after stimulus offset (0 s delay), repulsive sensory adaptation dominates (negative serial dependence). After 1 s of an imposed working memory delay, however, the effect flips to positive serial dependence, consistent with the human data. Serial dependence then increases gradually over time before reaching an asymptote within 10 s. (B) Peak-to-peak of the DoG fit across different ITIs. When the ITI is increased from 1 to 10 s, serial dependence becomes monotonically weaker until it is replaced by repulsive adaptation, again consistent with human behavioral results. (C) Tuning of adaptation across angular differences between consecutive stimuli for the 10-s ITI condition. The black trace depicts mean error, and the shading depicts the standard error of the mean. Although the magnitude of adaptation is weak (as in the human data), the repulsive deflection of errors is clearly present.

While these modeling results reproduce the scaling of serial dependence with both the length of the working memory delay period and of the ITI, they fall short of providing an explanation for the appearance of sensory adaptation in human behavior when there is no delay between stimulus and response and when the ITI between trials is as long as 10 s [15]. Adaptation is the quantitative opposite of serial dependence—a repulsion of the current percept away from the feature value of the previously held representation, tuned with the same DoG shape as positive serial dependence [1, 9, 15]. Unlike serial dependence, which has been linked to working memory, adaptation is believed to originate in early sensory networks [9]. To account for this phenomenon, we assumed that the input to our model of prefrontal cortex would be subject to adaptation, applied during feedforward sensory processing. We re-ran the full battery of simulations for Model 3 with the current injections that represent sensory inputs to the network shifted up to a few degrees relative to the previous stimulus’ feature value, in accordance with the DoG tuning function for adaptation. After this change, Model 3 reproduced the human behavioral results [15] throughout the full range of tested delay lengths and ITIs (Fig 4). Accounting for adaptation in this way did not improve the fit of the activity leak model to the behavioral data—serial dependence remained constant and positive at all the tested delay lengths and ITIs (S3 Fig).

## Discussion

In both the psychology [54–57] and neuroscience [22–25, 27] literatures, proposals for the organization of working memory have tended to be contentious rather than compatibilist. For example, cognitive scientists have debated for years whether distinct items in working memory are arranged in discrete slots or assigned continuous resources [54–57]. In this instance, modeling work has determined how neural circuits incorporate properties of both of these extremes [45], recasting the debate that pits the two theories against each other. The present study had a similar aim but in a different context. Our integrated model contains explicit computational elements of two major competing theories of working memory—synaptic reverberation to support elevated delay-period activity and synaptic plasticity to support “activity-silent” memory storage—and demonstrates in a novel way how the underlying mechanisms in these two models can operate synergistically to guide behavior. Previous investigations into the role of short-term plasticity in working memory have not considered the trial-history bias of serial dependence [22, 23, 26, 27, 33]. Here, we show that persistent activity drives synaptic weight changes that in turn can bias the drift of persistent activity on the subsequent trial. Although active firing and plasticity occur in tandem, their contributions to behavior can be dissociated. In our model, active firing is responsible for the maintenance of the working memory representation within the delay period of each trial, and synaptic augmentation is needed for the maintenance of the trace between trials. Without this division of labor—when the model is constrained such that active firing must be responsible for persistence of the memory trace both within and between trials—the fit to human behavioral data is poor.

The possibility remains that other combinations of active firing and synaptic plasticity mechanisms that we did not test provide an equivalently good fit to the dynamics of serial dependence observed empirically. For example, persistent elevated neural firing throughout the delay period—a tenet of the bump attractor model – may not be necessary for successful behavioral performance, especially if short-term plasticity occurs robustly enough for encoded feature values to be read out of the synaptic weights of the network via unstructured current injection [27]. Similarly, synaptic augmentation may not be necessary if other kinds of weight updates—such as depolarization-induced suppression of inhibition, mediated by prefrontal cannabinoids [58] – rise and fall in working memory networks with a comparable time course. The benefits of such refinements to our model remain to be demonstrated. What is clear from our collective modeling results, however, is that some amount of above-baseline active firing is needed in the networks that support working memory to propel synaptic plasticity at the magnitude and for the duration needed to explain the temporal dynamics of serial dependence. Traditional models that ignore plasticity and radical departures from conventional theory that ignore the importance of elevated active firing are unlikely to provide satisfactory accounts of the full range of biases typical of human cognition.

It is noteworthy that our synaptic augmentation model provides a quantitative (rather than merely qualitative) fit to the human behavioral data—matching both the time scale and amplitude of serial dependence [15]. In recent years, variations on the bump attractor model have settled for qualitative approximations of results from human psychophysics [45, 46], leading some to argue that, as a simplified model of cortex, it cannot make precise predictions about behavior [46]. Here, we show that even the reduced firing rate version of the model can be quite precise in its account of cognition. Other prominent working memory models that have yielded similarly close fits to behavior are typically agnostic about biophysical implementation [54, 59–64]. An exception is recent work by Paul Bays [65–67] that has delineated how Poisson-distributed spiking in a model of visual cortex implies particular deviations from normality in error distributions from continuous-report working memory tasks (like the one in Fig 1A). However, the equations of this model require dramatic violations of biophysical realism: synaptic dynamics are ignored, for example [65–67].

Combining neural modeling with behavioral and physiological experimentation in non-human primates, Papadimitriou and colleagues have attempted to uncover the neural mechanisms of serial dependence in working memory [17, 18]. Similar to our approach, they constructed their neural model iteratively, establishing the insufficiency of simpler, alternative versions to account for the full range of empirical findings. However, as a result, their final model attains explanatory power at the expense of parsimony. It requires that multiple independent memory storage sites with different decay rates combine to guide behavior [17], that receptive fields shift with a particular magnitude as a function of recent experience [18], and that Hebbian plasticity in projections to a downstream “readout” circuit causes a reversal of the population response before the motor output is generated [18]. While the model is a good fit to monkey behavior, these assumptions go beyond what physiological data have demonstrated is plausible, and the biophysics in their final model is incompletely specified: the receptive field shifts and Hebbian plasticity are applied instantaneously via hard-coding of values. Our model, in contrast, achieves a comparable fit to human behavior with a single population of neurons, undergoing synaptic updates with precisely the same temporal dynamics that have been measured physiologically in prefrontal cortex [38, 39].

One potential criticism of our augmentation network model is that it assumes that all synapses in the circuit experience the same kind of plasticity, which is demonstrably false in real cortical networks [38, 39]. However, it is at this level of analysis that the realism of the bump attractor model as a whole breaks down. The reduced version of the model we used as our starting point contains just 256 neurons [44], far fewer than would be expected to participate in the performance of a working memory task *in vivo*. Hence, these neurons should be viewed as abstractions of a larger collection of cells with heterogeneous synaptic dynamics, but for which the augmentation signal is prominent [38, 39]. From this perspective, the small amplitude of augmentation in individual synapses in our network (see Materials and methods) should be interpreted as the average over many synapses, only some of which are reliably plastic. This consistent scaling of magnitudes solidifies, rather than detracts from, the biophysical accuracy of our model. In the past, the bump attractor model has been found to perform equivalently regardless of whether heterogeneity among cells is explicitly coded, as long as homeostatic processes keep connections balanced [68].

## Materials and methods

### General description of the model

All model simulations were performed with a working memory task that we depict as testing memory for the stimulus’ angle from fixation (Fig 1A). All of the code used to perform the simulations was written using the Python package brian2 [69] and is available in a public Git repository (https://github.com/dabliss/bliss_sd_model_2017). We used the reduced firing-rate version of the bump attractor model that has been demonstrated to recapitulate the activity patterns of the full, spiking version [32, 44, 52].

In this model, excitatory signaling for each neuron is defined with a single variable *s* that represents NMDA conductance (specifically, the fraction of open channels). In the original spiking version of the model, explicit equations were included for signaling at AMPA and GABA_A_ receptors as well [32]. However, because synaptic gating at these receptor types has a much shorter time constant (∼2 ms) than at NMDA receptors (∼100 ms), the model can be simplified to exclude them without disrupting network stability during the delay period [52]. (In contrast, NMDA receptors are necessary for stability in the model [30, 32, 52], and this necessity of NMDA receptors for stable delay-period activity in prefrontal cortex has been confirmed empirically [48].) The differential equation for *s* is

$$\begin{array}{c} \frac{ds}{dt}=-s/\tau_{s}+\left(1-s\right)\gamma f\left(I\right), \end{array}$$ (1)

with *γ* = 0.641 and *τ*_*s*_ = 60 ms. The firing rate *f*(*I*) for each neuron is a function of the total synaptic current *I*:

$$\begin{array}{c} f\left(I\right)=\frac{aI-b}{1-\exp[-d(aI-b)]}, \end{array}$$ (2)

with *a* = 270 Hz/nA, *b* = 108 Hz, and *d* = 0.154 s. The total synaptic current comes from three sources: recurrent signaling, sensory drive, and random noise (*I* = *I*_*r*_ + *I*_*s*_ + *I*_*n*_).

The network is fully connected, meaning that each neuron *i* receives recurrent input from every other neuron *j* in the network. This input is summed as follows:

$$\begin{array}{c} I_{r,i}=\sum_{j}g_{ij}s_{j}, \end{array}$$ (3)

where *g*_*ij*_ is the synaptic coupling from *j* to *i*.

The connection strengths are not uniform, but depend on the tuning of individual neurons. Neurons in the network are tuned to the task-relevant stimulus feature, and collectively their tuning curve centers uniformly tile the range of possible preferred stimulus angles (from 0-360°). The original spiking version of the network contained thousands of neurons [32]. However, in the reduced version of the model used here, 256 neurons is sufficient to achieve stable network performance [44]. Hence, we modeled just 256 neurons for each of our simulations. The synaptic couplings *g*_*ij*_ throughout the network have a Gaussian profile over all possible differences in tuning between neurons with preferred angles *θ*_*i*_ and *θ*_*j*_:

$$\begin{array}{c} g_{ij}\left(\theta_{i}-\theta_{j}\right)=J_{-}+J_{+}\exp\left(-{\left(\theta_{i}-\theta_{j}\right)}^{2}/2\sigma^{2}\right). \end{array}$$ (4)

For Models 1 and 2, *σ* = 43.2°. For Model 3, *σ* = 50°, except in the version used to generate the orange tuning curve in S2 Fig, for which *σ* was set to 30°. Parameters *J*_−_ and *J*_+_ determine the levels of recurrent inhibition and excitation in the network, respectively. These levels are balanced such that the population can sustain persistent firing. For Models 1 and 2, we used *J*_+_ = 2.2 nA and *J*_−_ = −0.5 nA. For Model 3, we used *J*_+_ = 1.52 nA and *J*_−_ = −0.5 nA, except in the version with narrow tuning curves, where *J*_+_ = 2.35 nA.

When a stimulus at a particular angle *θ*_*s*_ is presented at the start of each trial of the task, neurons receive an injection of sensory-driven current that depends on their preferred angle *θ*:

$$\begin{array}{c} I_{s}=g_{s}\exp\left(-{\left(\theta_{s}-\theta \right)}^{2}/2\sigma_{s}^{2}\right), \end{array}$$ (5)

where *σ*_*s*_ = 43.2° and *g*_*s*_ = 0.02 nA. In all simulations, stimuli were presented for 1 s (as in [15]).

For versions of the model with the stimulus subject to sensory adaptation, we shifted *θ*_*s*_ for the second trial in each trial pair as a function of the difference between its value and that of the first trial. This shift was computed using the DoG function (see below) with parameter values *a* = −0.015 and *w* = 0.6 for an ITI of 1 s. We assumed that adaptation decays exponentially as the ITI is lengthed. The time constant of decay that provided the best fit to human behavioral data [15] was 5.592 s.

Random noise passed into the network (*I*_*n*_) represents background activity in cortex unrelated to the task. Noise varies over time as

$$\begin{array}{c} \tau_{n}dI_{n}/dt=-\left(I_{n}-I_{0}\right)+\sqrt{\tau_{n}}\sigma_{n}\eta \left(t\right), \end{array}$$ (6)

where *η*(*t*) is white Gaussian noise, *I*_0_ = 0.3297 nA, *τ*_*n*_ = 2 ms, and *σ*_*n*_ = 0.009 nA.

We decoded the center of the activity bump to compute a behavioral response from the network during the delay period of each trial using the population vector method [32, 51].

### Activity leak mechanism for Model 2

In Model 1, the activity bump is reset during a response period that lasts 300 ms, throughout which unstructured inhibitory current (−0.08 nA) is passed into the circuit [70]. In order to allow a residual bump of activity to persist into the ITI, at a magnitude consistent with what has been observed in monkey cortex [18], we changed the reset signal to be −0.00925 nA. For the version of the model in which activity was allowed to extend into the ITI unabated, we set the reset signal to 0 nA.

### Plasticity rule for Model 3

Our implementation of synaptic augmentation is based on [50]. We define the synaptic vesicle release probability *F* for each synapse as

$$\begin{array}{c} \frac{dF}{dt}=\alpha \left(x-F\right)f\left(I\right)-\frac{F}{\tau_{F}}, \end{array}$$ (7)

where *α* = 0.015, *x* = 0.008, and *τ*_*F*_ = 4.2 s—which matches the best-fitting time constant for measurements of synaptic augmentation in prefrontal cortex [39]. Theoretically, *F* is bounded between 0 and 1, and the value of *x* used here ensures that *F* stays at the lower end of this range. In addition, the synapses in Model 3 were susceptible to synaptic depression. This requires a differential equation for the fraction of available vesicles *D*:

$$\begin{array}{c} \frac{dD}{dt}=-pf\left(I\right)FD+\frac{1-D}{\tau_{D}}, \end{array}$$ (8)

where *p* = 0.01 and *τ*_*D*_ = 1 s.

The equation for the NMDA conductance was updated to

$$\begin{array}{c} \frac{ds}{dt}=-s/\tau_{s}+\left(1-s\right)\gamma \left(y+F\right)Df\left(I\right), \end{array}$$ (9)

where *y* = 0.992. (Strictly, the total vesicle release probability for each synapse in the model is *F* + *y*, with *y* fixed. This sum is always between 0 and 1.)

In the version of Model 3 with the stimulus input subject to adaptation, we adjusted some parameter values as follows: *τ*_*F*_ = 3.8 s, *p* = 0.006, *x* = 0.014, and *y* = 0.986.

### Simulations

To measure serial dependence in responses from the three models, we ran trials back-to-back in pairs. The connections of the network are radially symmetric, allowing us to place the first stimulus in each trial pair at 180° without loss of generality. Thirty-two angles were tested for the second stimulus of each pair, evenly spaced between 0 and 360°. For each of these 32 pairs, 100 simulations were run using different random seeds.

The timing of task events matched the protocol used to characterize the time course of serial dependence in human behavior [15]. Specifically, each stimulus was presented for 1 s, and a 1-s ITI was used. To conserve computational resources, we set the first trial in each pair for all simulations used to generate our final results to have a delay period of 1 s. The second trial in each pair had a delay period of 10 s, and the behavioral response was decoded at several time points within the delay (0, 1, 3, 6, and 10 s [15]). Decoding took as input a window of population activity starting 100 ms before the time point of interest.

For Model 3, we ran additional batteries of simulations in which the ITI was varied. Again, 100 simulations (with different random seeds) were run for sets of trial pairs that spanned the circular range of stimulus differences and for each of four ITIs (1, 3, 6, and 10 s [15]).

### Characterization of serial dependence

We used the derivative of Gaussian (DoG) to characterize the tuning of serial dependence across all possible differences between past and current visual input. This function has been used in several other studies for this purpose [1, 2, 8, 9, 15]. The DoG is defined as

$$y=xawce^{-{\left(wx\right)}^{2}},$$ (10)

where *y* is the signed error, *x* is the relative angle of the previous trial, *a* is the amplitude of the curve peaks, *w* is the width of the curve, and *c* is the constant $\sqrt{2}/e^{-0.5}$. We used the scipy [71] function least_squares (in the optimize module) to find the values of *a* and *w* that minimized the difference, for each *x*, between the estimated *y* and each model’s errors. Across all values of *x*, we take the magnitude of serial dependence to be the peak-to-peak of *y*(*x*), with the sign adjusted to match the direction of the effect (positive if the response is attracted towards the previous stimulus, negative if repelled from it).

We computed bootstrapped confidence intervals for the serial dependence magnitude in each condition as follows [2, 9]: We resampled the data with replacement 10,000 times. To each resampled dataset, we fit the DoG. This yielded a distribution of peak-to-peak values from which we selected the boundaries of the 95% confidence interval.

## Supporting information

S1 Fig Results for Model 2 with response-period current set to zero. (A) Tuning of serial dependence across angular differences between consecutive stimuli when the current trial’s delay period length is 0 s. Raw errors are in gray. The DoG function provides a poor fit to this pattern and hence is omitted. Decoded responses from the network tend to cluster around the previous trial’s stimulus value, and information for the current trial’s stimulus is lost. (B) Tuning of serial dependence across angular differences between consecutive stimuli when the current trial’s delay period length is 10 s. Raw errors are in gray. The pattern of serial dependence in this version of the model does not change as the delay length is extended from 0-10 s.
(EPS)

S2 Fig Results for Model 3 with varied tuning widths of individual neurons. Tuning of serial dependence across angular differences between consecutive stimuli when the width of individual neurons’ tuning curves is *σ* = 30° (orange) or *σ* = 50° (blue). The central line in each trace depicts mean error, and the shading depicts the standard error of the mean. Wider tuning curves in the network produce a wider (and larger amplitude) pattern of serial dependence.
(EPS)

S3 Fig Results for Model 2 after accounting for adaptation. Peak-to-peak of the DoG fit across the working memory delay. Shading represents bootstrapped 95% confidence intervals. Immediately after stimulus offset (0 s delay), positive serial dependence is already present in responses. The magnitude of this serial dependence does not change as the delay is lengthened to 10 s.
(EPS)
